# Target splitting in radiation therapy for lung cancer: further developments and exemplary treatment plans

**DOI:** 10.1186/1748-717X-4-30

**Published:** 2009-08-14

**Authors:** Karl Wurstbauer, Heinz Deutschmann, Peter Kopp, Florian Merz, Helmut Schöller, Felix Sedlmayer

**Affiliations:** 1 Department of Radiation Oncology and radART – Institute for Research and Development on Advanced Radiation Technologies at the Paracelsus Medical University, Salzburg, Austria

## Abstract

**Background:**

Reporting further developments evolved since the first report about this conformal technique.

**Methods:**

Technical progress focused on optimization of the quality assurance (QA) program, especially regarding the required work input; and on optimization of beam arrangements.

**Results:**

Besides performing the regular QA program, additional time consuming dosimetric measurements and verifications no longer have to be accomplished.

'Class solutions' of treatment plans for six patients with non-resected non-small cell lung cancer in locally advanced stages are presented. Target configurations comprise one central and five peripheral tumor sites with different topographic positions to hilus and mediastinum. The mean dose to the primary tumor is 81,9 Gy (range 79,2–90,0 Gy), to macroscopically involved nodes 61,2 Gy (range 55,8–63,0 Gy), to electively treated nodes 45,0 Gy. Treatments are performed twice daily, with fractional doses of 1,8 Gy at an interval of 11 hours. Median overall treatment time is 33 days. The set-up time at the linac does not exceed the average time for any other patient.

**Conclusion:**

Target splitting is a highly conformal and nonetheless non-expensive method with regard to linac and staff time. It enables secure accelerated high-dose treatments of patients with NSCLC.

## Background

In order to improve locoregional tumor control of lung cancer patients by radiation therapy, raising of the tumor dose is mandatory. This constitutes a challenge to be overcome only by the use of conformal, healthy tissue sparing techniques. Following rather simple 3D approaches, sophisticated forms of intensity modulated techniques such as tomotherapy, intensity modulated arc therapies or volumetric modulated arc therapies have been described recently and begin to be applied clinically [1,2]. Results of treatments of lung cancer patients with these latter techniques are still missing.

In 1999 our first report about the conformal technique of target splitting in external radiotherapy of lung cancer has been published [3]. Since then, we use this method routinely for lung cancer patients in all stages. During the past years, this technique has continuously been evolved with regard to optimizing the procedures for quality assurance and raising conformity of the treatment plans.

**Figure 1:**
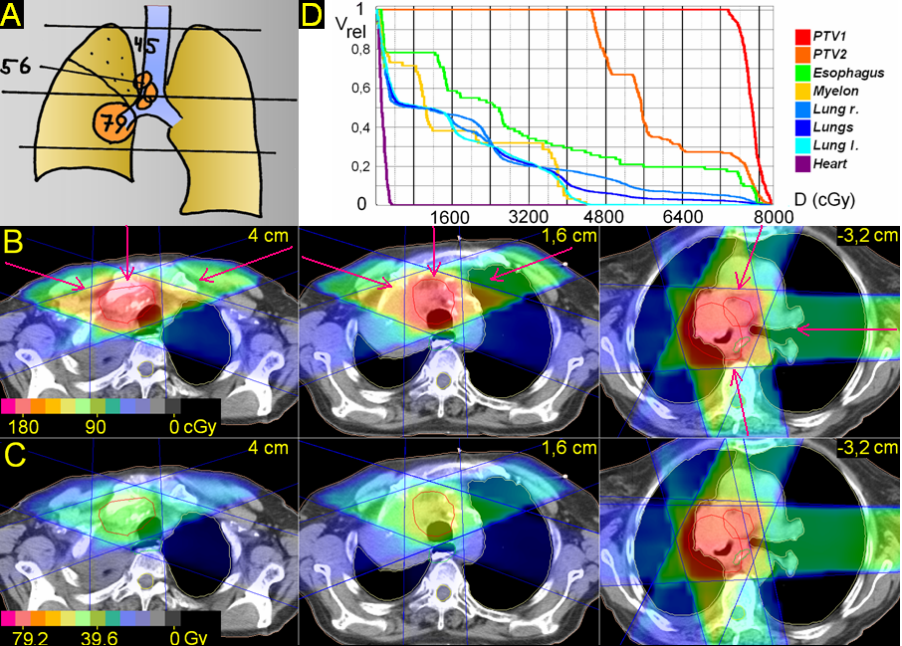
**Centrally located tumor**. 83 years; central squamous cell carcinoma, 4 cm ∅, atelectasis upper lobe, paralysis phrenical nerve with elevated diaphragma; enlarged PET-positive ipsilateral mediastinal nodes. A. Scheme; position of junction plane and upper and lower borders, doses (Gy). B. Treatment plan single fraction. C. Overall treatment plan. D. DVHs.

**Figure 2:**
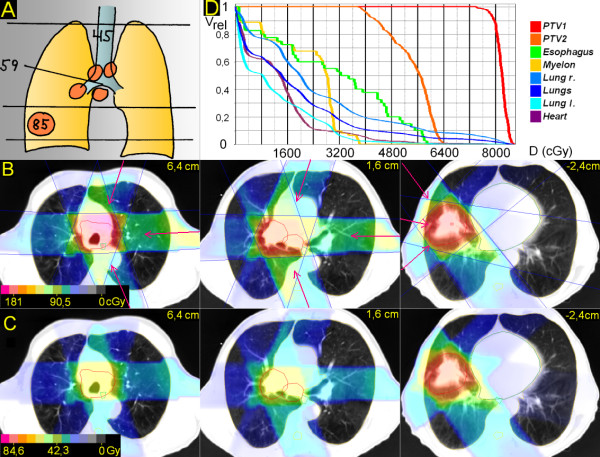
**Peripheral tumor, hilus/mediastinum to be treated not within the craniocaudal extension of the the primary tumor**. 53 years; squamous cell carcinoma peripheral lower lobe, 5,5 cm ∅; enlarged PET-positive hilar, subcarineal and bilateral mediastinal nodes. A. Scheme; position of junction plane and upper and lower borders, doses (Gy). B. Treatment plan single fraction. C. Overall treatment plan. D. DVHs.

**Figure 3:**
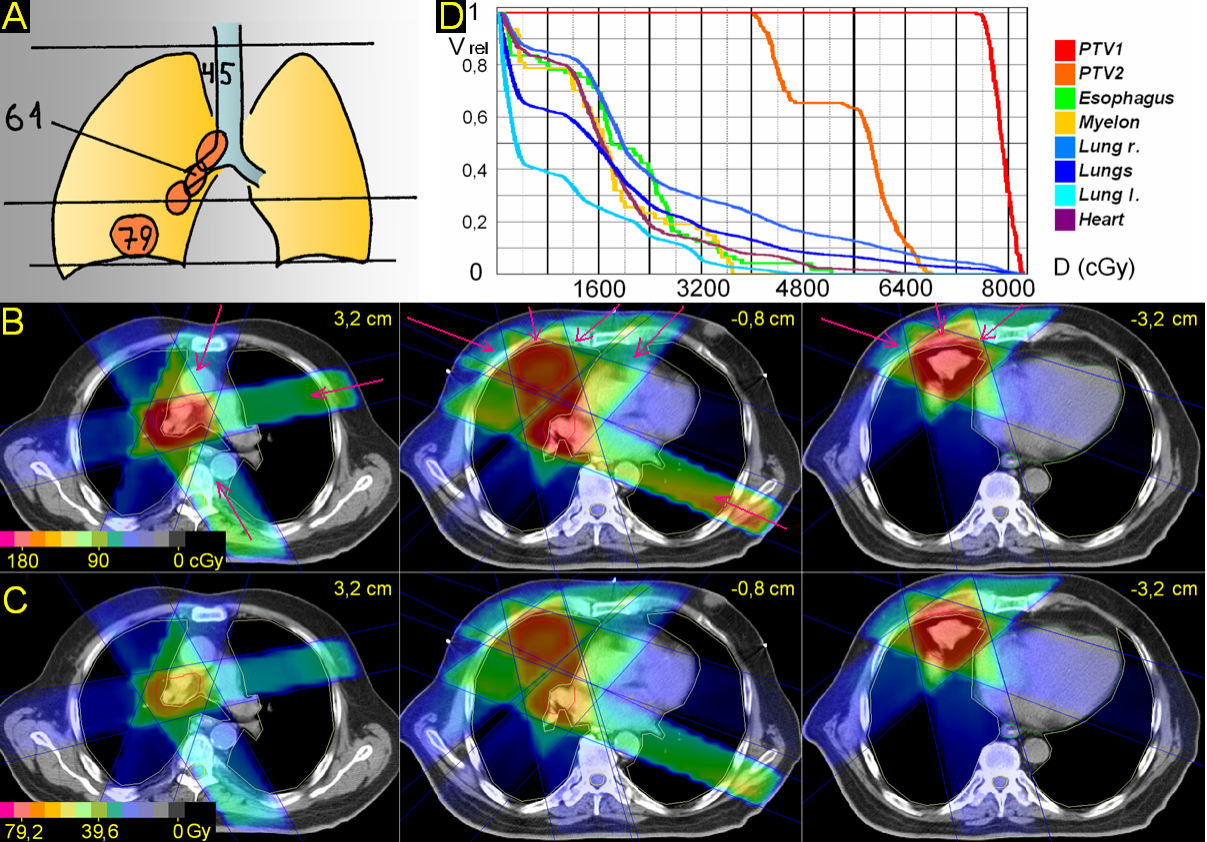
**Peripheral tumor, hilus/mediastinum to be treated partially within the craniocaudal extension of the the primary tumor**. 73 years; squamous cell carcinoma basal middle lobe, 4,2 cm ∅; enlarged PET-positive hilar and ipsilateral mediastinal nodes. A. Scheme; position of junction plane and upper and lower borders, doses (Gy). B. Treatment plan single fraction. C. Overall treatment plan. D. DVHs.

**Figure 4:**
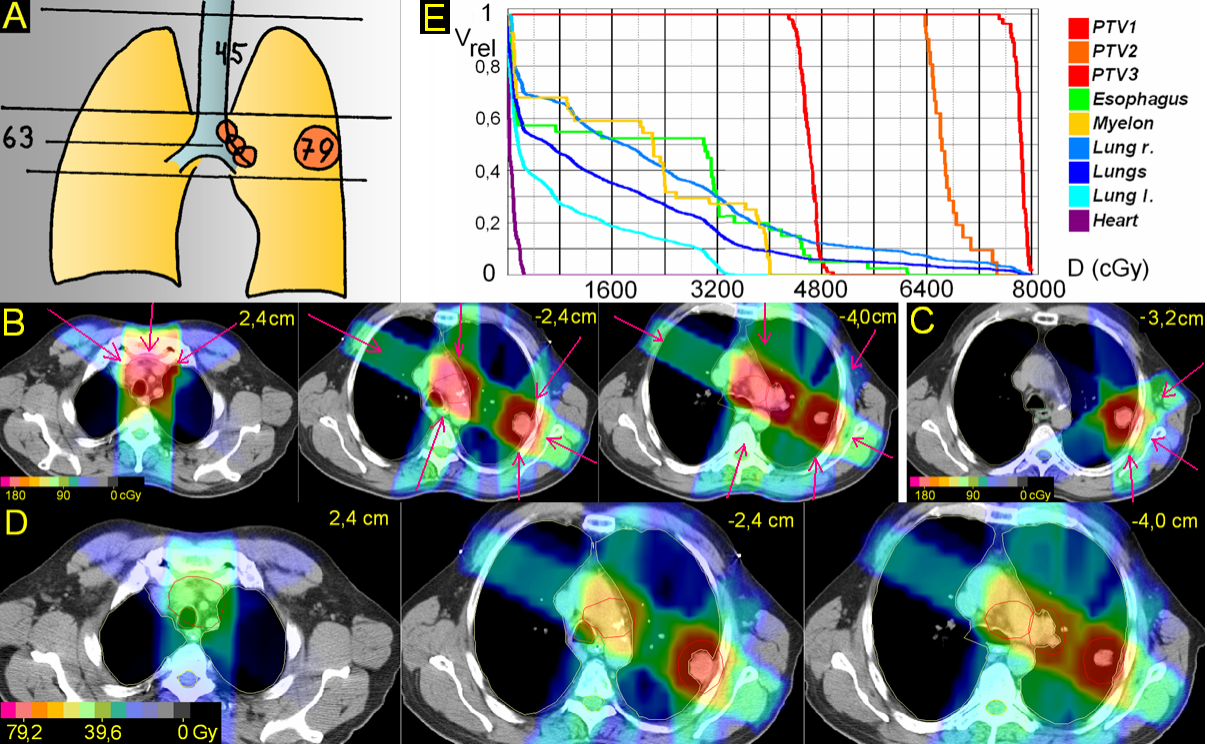
**Peripheral tumor located lateral and distant to hilus/mediastinum**. 48 years; squamous cell carcinoma peripheral upper lobe, 4 cm ∅; enlarged PET-positive hilar and ipsilateral mediastinal nodes. A. Scheme; position of junction plane and upper and lower borders, doses (Gy). B. Treatment plan single fraction. C. Treatment plan single fraction of boost to primary tumor. D. Overall treatment plan. E. DVHs.

**Figure 5:**
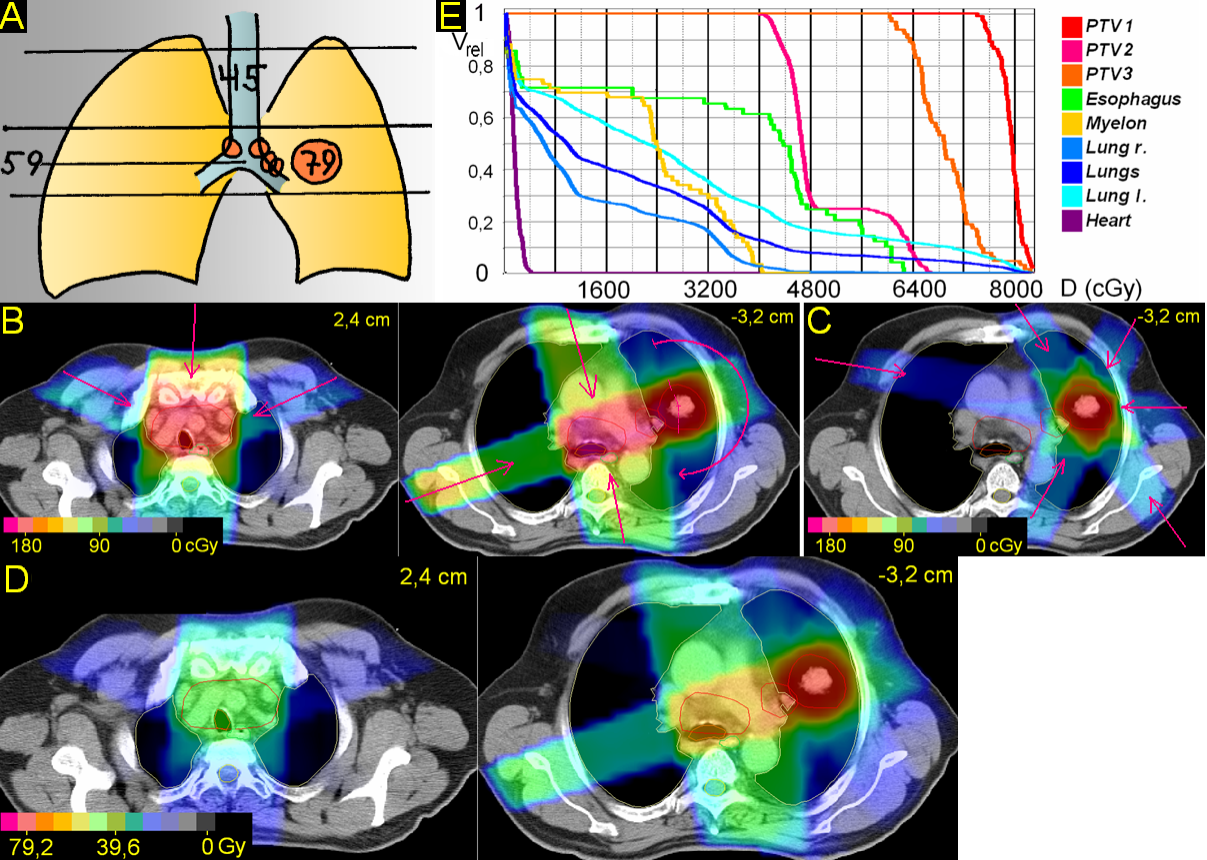
**Peripheral tumor located lateral, but close to hilus/mediastinum**. 67 years; adenocarcinoma peripheral upper lobe, 3,5 cm ∅; enlarged hilar nodes, mediastinoscopically proven bilateral mediastinal nodes. A. Scheme; position of junction plane and upper and lower borders, doses (Gy). B. Treatment plan single fraction. C. Treatment plan single fraction of boost to primary tumor. D. Overall treatment plan. E. DVHs.

**Figure 6:**
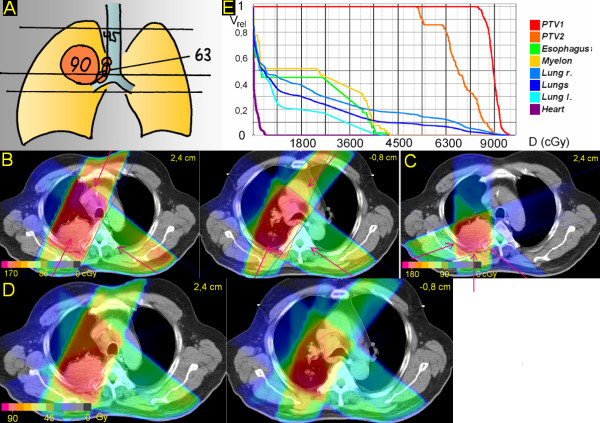
**Peripheral tumor, junction plane set within the primary tumor**. 62 years; squamous cell carcinoma dorsal upper lobe with infiltration of the chest wall, 6,5 cm ∅; enlarged PET-positive hilar and ipsilateral mediastinal nodes. A. Scheme; position of junction plane and upper and lower borders, doses (Gy). B. Treatment plan single fraction. C. Treatment plan single fraction of boost to primary tumor. D. Overall treatment plan. E. DVHs.

This report gives an update about the technical innovations and implications for workflow and demonstrates exemplary treatment solutions in 6 lung cancer patients with different tumor topographies (Figure 1, Figure 2, Figure 3, Figure 4, Figure 5 and Figure 6).

## Methods

The technique of target splitting has been described in detail [3]. In an individually chosen transversal plane, the target is split into a cranial and a caudal part. For either part completely independent beam arrangements are designed. Half collimated, coplanar asymmetric fields ('half beams'), in general each adjacent to the isocentric splitting (junction) plane, allow for set-up of highly conformal treatment plans.

### Progress in Quality Assurance (QA) since the first report

**Figure 7:**
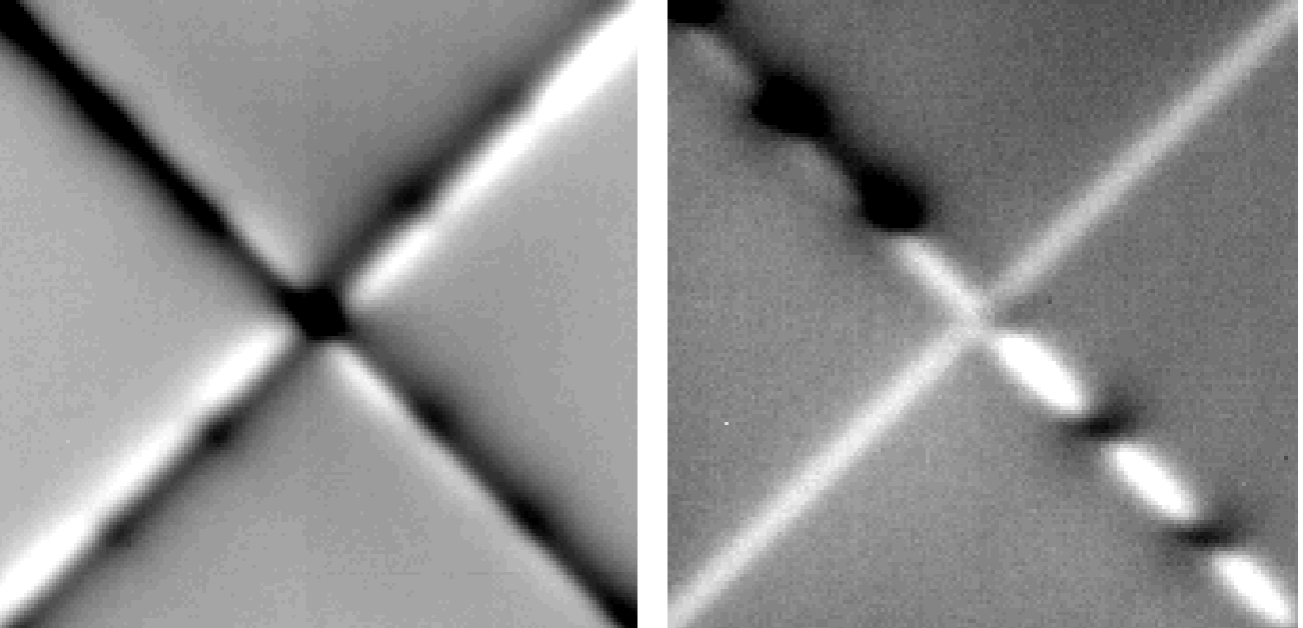
**Dosimetric verification of accuracy of field junctions of a MLC head**. White and black levelled regions represent dose inhomogeneities below 10%. Left: One double half collimated quadrant beam (45°) was irradiated 4 times with relative intersegmental collimator rotations of 0°(I), +90°(II), +180°(III), -90°(IV). Right: 4 segments, each irradiated in different quadrants (I-IV) by changing the field aperture with a fixed collimator rotation (45°).

In order to prevent over- or underdosages in or next to the junction plane, special care has to be taken to ensure correct positioning of independent jaws at the central axis. As an individual fine adjustment of MLC jaws for each patient is time consuming, we developed and implemented a QA program which is periodically testing for over- or underdosages by means of amorphous silicon flat panel imaging (EPID). Because different collimator rotations (0°, ± 90°) will be applied in clinical cases for optimal MLC coverage and/or to allow the insertion of a motorized wedge, all combinations of possibly adjacent jaws (x1/x2, x1/y1, x1/y2, y1/x2 and y1/y2) have to be tested. On a monthly basis and after each head maintenance, five different sequences of beam segments are irradiated onto the panel: the first four sequences deliver each one quadrant field with four intersegmental collimator rotations (0°, 90°, 180°, -90°) to be summed up in one image per sequence. The last sequence keeps the collimator rotation at 0°, while irradiating the four quadrants by changing the jaw- (and leave) positions (Figure 7).

This method inherently guarantees that all jaw-offsets will be aligned to the radiation field's central axis as defined by the mechanical axis of collimator rotation. If over- or underdosages are measured along the junction lines, a straight forward calibration of jaw and leave positions with sub-millimeter accuracy is possible, if the relationship between maldosage and field-shift is known. The latter can easily be determined once in advance from single jaw and MLC-leaf penumbra measurements. However, in detail, the problem has some degree of complexity, since the relative position of a leaf to the closely following backup-jaw will influence the gradient of the penumbra as well as inter-leave-leakage in the junction plane. Additionally, different penumbra gradients of x and y jaws (due to their different distance to the focus of the machine) will sum up to an unavoidable, slightly inhomgeneous dose distribution apparent as parallel regions of over- and underdosage next to the junction plane (Figure 7). Over- or underdosages in the range of up to 10% within a zone of less than ± 1 mm can be neglected. Although this error might be increased in principal by connecting two opposing beams (knowing that the machine's isocenter is a sphere or ellipsoid with radii in the range of 1 mm rather than a point), patient's daily setup deviations and intrafractional respiratory movements will blur the overall maldosage in the junction plane as well as distributed gantry- and collimator angles, which has been shown in a series of phantom-film measurements.

### Patient set-up, planning procedure and treatment delivery

Six exemplary treatment plans which cover different target volume constellations have been chosen among patients with advanced-stage NSCLC treated in the past three years. All six patients participate in a prospective study, in which the dose to the primary tumor is correlated to its size [4]. Two patients are staged T2N2 and T2N3, respectively; one patient T3N2 and T4N2, respectively.

Patients are set up in vacuum cradles, usually supine with the hands above the head. A planning CT in treatment position is performed as 'slow CT' from the apex to the bases of the lung, patients freely breathing (non-spiral CT; 4 s/slice; slice thickness 7 mm formerly, more recently 5 mm; couch movements 8 mm or 5 mm) [5]. In the case of atelectasis 18-fluorodeoxyglucose positron emission tomography (FDG-PET) is performed in treatment position and the slices are matched with the planning CT. Margins from gross tumor volume (GTV) to planning target volume (PTV) are 7 mm, regarding primary tumor, macroscopically involved lymph nodes and elective lymph node stations, defined as the region about 5 to 6 cm cranial to macroscopically involved nodes. In contouring of the organs at risk, the GTV is excluded from the lung volume, the heart is contoured from about 1 cm below the level where the lower edge of the pulmonary trunk crosses the median to the apex of the heart. Esophagus and spinal cord are contoured in their entire thoracic length.

Planning is performed with a 3D-planning system (*Oncentra Masterplan*), inhomogeneities are taken into account by a pencil beam algorithm. Dose constraints for the spinal cord were set at 45 Gy, V20 (volume receiving >20 Gy) for a single lung at 50%, V25 for both lungs considered as a single organ at 30%, the maximal dose to the esophagus at 80 Gy (measured in the center of the esophagus at its most exposed level).

Treatments were delivered with 15 MV photons, fractional doses of 1,8 Gy (ICRU), twice daily, interval 11 h.

Since 2006, daily image guidance (IGRT) was performed by MV cone beam CTs, since 2007 by orthogonal kV-imaging with adjustment to the esophageal and large airways' structures [6].

## Results

In contrast to our previous report about this technique, besides performing the regular QA program as described, no additional time consuming dosimetric verifications have to be accomplished.

For all 6 patients treatment plans with one single isocenter can be provided. This enables a remote control of all treatment steps by assisted setup functions. The daily set-up time at the linac does not exceed the average time for any other patient.

### 1. Centrally located tumor (Figure 1)

The junction plane is chosen above the central tumor. The upper volume is treated by anterior-posterior (a-p) – right oblique anterior and left oblique anterior, partially wedged beams (290°, 0°, 70°), the lower volume by left oblique anterior, left lateral and left oblique posterior, partially wedged beams (25°, 90°, 165°). After 45 Gy (elective dose for not macroscopically involved nodes) the upper jaws of the upper volume are closed asymmetrically for a length of 5 cm. After 55,8 Gy (dose for macroscopically involved nodes) the primary tumor is boosted to 79,2 Gy (excluding the nodes by setting of MLCs). V20 of the right lung is 43%, of the left lung 32%, V25 for both lungs 28%. In the upper volume the esophagus can be spared very well; in the lower volume, because the primary tumor is partially directly adherent to the esophagus, for about 3 cm it receives the full tumor dose at a major part of its circumference.

### 2. Peripheral tumor, hilus/mediastinum to be treated not within the craniocaudal extension of the the primary tumor (Figure 2)

The junction plane is chosen above the primary tumor, below the hilus. The upper volume is treated by 3 partially wedged, left-sided beams (20°, 90°, 160°) to 59,4 Gy; after 45 Gy the upper jaws are retracted asymmetrically for 5,5 cm. The lower volume (primary tumor) is treated with three partially wedged right-sided beams (320°, 280°, 220°) to 84,6 Gy. V20 right and left lung and V25 both lungs is 37%, 26% and 27%, respectively. V50 for the heart is 2%.

### 3. Peripheral tumor, hilus/mediastinum to be treated partially within the craniocaudal extension of the the primary tumor (Figure 3)

The junction plane is chosen above the primary tumor. The upper volume (hilus and mediastinum) is treated by three, partially wedged fields (20°, 80°, 150°) to 61,2 Gy. After 45,0 Gy the upper volume is reduced cranially for 6 cm. In the lower volume, the primary tumor is treated by three, partially wedged fields (290°, 345°, 50°); only one of these fields (345°) meets also the PTV of the nodes, situated only in the upper 2 cm of the caudal volume; the missing dose is supplied by two partially wedged fields (45°, 115°), which do not interfere grossly with the PTV of the primary tumor. After 61,2 Gy these two fields are withdrawn and the primary tumor alone is treated to 79,2 Gy. V20 right and left lung and V25 both lungs is 49%, 22% and 26%, respectively. V50 for the heart is 2%.

### 4. Peripheral tumor located lateral and distant to hilus/mediastinum (Figure 4)

The junction plane is chosen above the primary tumor. The upper volume (elective nodes only) is treated by three, partially wedged beams (305°, 0°, 55°) to 45 Gy. Within the lower volume, the primary tumor is treated by four, partially wedged fields (35°, 120°, 180°, 300°). Two of these fields (120°, 300°) meet also the PTV of the nodes, the missing dose to the nodes is added by two fields, which do not interfere with the primary tumor. After 63,0 Gy the primary tumor alone is boosted to 79,2 Gy. V20 left and right lung, V25 both lungs is 47%, 16% and 26%, respectively. In the upper volume the esophagus could have been better spared chosing a less steep angle for the oblique beams (e.g. 285° and 75° instead of 305° and 55°). However, as the whole upper volume is treated only with an elective dose (45 Gy), significant esophageal side effects have not been observed, and the beam angles were optimized with regard to sparing of lung tissues. In the lower volume the esophagus can be spared fairly. D(max) for the heart is 3,5 Gy.

### 5. Peripheral tumor located lateral, but close to hilus/mediastinum (Figure 5). Junction plane above the primary tumor

The isocenter is set in the center of the primary tumor, which is treated by a rotational arc (345° to 180°) and a right sided field (250°). The missing dose to the hilus and mediastinum is added by two partially wedged fields (335°, 170°). After the dose to the nodes is reached (59,4 Gy), the primary tumor is boosted by an arrangement of six fields (25°, 85°, 145°, 205°, 265°, 325°) to 79,2 Gy. Due to histological proof of bilaterally positive nodes in the middle mediastinum, the whole upper mediastinum has been electively irradiated up to 45 Gy (by three partially wedged fields; 290°, 0°, 70°). V20 left and right lung, V25 both lungs: 52%, 32% and 32%, respectively. D(max) to the heart: 5 Gy.

### 6. Peripheral tumor, junction plane set within the primary tumor (Figure 6)

In order to optimize the angles of the beam arrangements the junction plane is set within the primary tumor. The upper volume is treated by oblique opposing plus left oblique beams (25°, 125°, 205°), with good sparing of spinal cord and esophagus, at the cost of some medial parts of the right lung. In the caudal volume, the oblique ventral beam can be taken less steep (40°, 125°, 200°), resulting in a better sparing of the lung while maintaining good sparing of myelon and esophagus,. This series is treated to 63,0 Gy; after 45 Gy the elective nodes of the upper mediastinum are withdrawn by setting of MLCs. In a second series the primary tumor is boosted to 90,0 Gy (130°, 180°, 250°). V20 right and left lung and V25 both lungs is 37%, 19% and 25%, respectively. D(max) for the heart is 6 Gy.

The mean dose to the primary tumor of these six patients amounts to 81,9 Gy (79,2 – 90,0 Gy), to macroscopically involved nodes 61,2 Gy (55,8 – 63,0 Gy), and to elective nodes 45,0 Gy in an accelerated fractionation schedule. The median overall treatment time was 33 days (31 – 38 days).

## Discussion

In primary radiation therapy of NSCLC a positive dose-response relationship with regard to tumor control and survival seems to be proven [7,8]. Furthermore, in order to prevent accelerated repopulation of clonogenic tumor cells, a short overall treatment time is important [9,10]. In this study we present exemplary treatment plans of patients with different topographical realities, in which doses up to 90,0 Gy in 33 days have been safely applied. Thereby beam arrangements are shown, which to our knowledge have not been published previously.

In 1979 Williamson first described the matching of orthogonal fields by an isocentric half-beam technique, using a large lead block positioned in the accessory tray at the beam axis [11]. He proposed this method for head and neck, breast and craniospinal treatments. With the availability of independently moving jaws asymmetric collimators were used to split the beam for head and neck patients [12]. In 1999, in our previous report we proposed this technique not only for matching orthogonal fields, but to perform completely independent planning and treatments on both sides of the junction plane, including rotational elements, static fields at arbitrary angles, wedge filters, etc. [3]. We called this technique 'target splitting', because the positioning of the junction plane depends on shape and topographic parameters of the target and its surroundings.

The method was initially applied to lung cancer patients. With ongoing practice some 'rules' evolved, breaking some former "taboos" in radiotherapy of lung cancer:

1. Minimizing the dose to the ipsilateral (i.e. tumor bearing) lung.

In many cases the ipsilateral lung will be the first organ to reach the dose constraint. This can be avoided by setting beams via median structures (spine, anterior mediastinum), mostly angled to the contralateral lung (e.g. caudal volume of patient 1). The contralateral lung is irradiated if necessary to its tolerance limit.

2. If necessary, for optimizing beam arrangements junction planes can easily be set within the primary tumor itself (e.g. patient 6) or within macroscopically involved nodes (e.g. patient 1, 4, 6) (comments below).

As to elective nodal irradiation, usually the region about 5 – 6 cm above macroscopic nodal disease is included into the PTV. If the upper mediastinal nodes are involved, a supraclavicular field is used. Most studies engaged in dose escalation of NSCLC disapprove elective nodal irradiation, in order to gain potential to raise the dose to the primary tumor [8,13]. However, isolated elective nodal recurrence occurs. Rosenzweig et al describe an actuarial elective nodal failure rate at 2 years in locally controlled patients of 9% [14]. RTOG 9311, also omitting elective nodal irradiation, reports 12/176 patients with isolated elective nodal recurrences [13]. Microscopic spread cranial to macroscopically involved nodes must be assumed in a relevant portion of patients and a 'collateral' dose from the macroscopic PTVs in these sites is not applied. Because FDG-PET scans detect malignant tissue only at a minimal size of about 0,5 cm, this mode has been retained unchanged also with the availability of PET staging. In our experience of treating >100 patients with 45 Gy in 2,5 weeks, no isolated recurrence in electively treated sites until now has been observed.

Regarding pulmonary doses, when we started to implement target splitting and to raise the dose, we set the constraints as recommended for safe 3D-treatments some years ago: a dose of ≥ 20 Gy should not exceed 50% of the volume of a single lung and ≥ 25 Gy should not exceed 30% of the volume of both lungs together [15-17]. Observing these limits resulted in a high tolerability using the target splitting technique. However, patients with preexisting lung fibrosis should be excluded from accelerated, high dose therapies [4]. With regard to the esophagus we limited the maximum dose in accelerated schedules to 80 Gy. Such a high dose rarely must be applied because the esophageal dose mostly is determined by the dose given to the nodes, not to the primary tumor and because target splitting has a capability also to spare the esophagus. In our experience of 15 years with high dose treatments of lung cancer patients we did not observe any severe late esophageal toxicity [4,18,19].

To account for sufficient margins, a rim of 7 mm from GTV to PTV in patients freely breathing might appear rather tight. This issue has been described and discussed in detail previously [5]. Shortly, slow planning CTs depict the different relevant positions of the moving tumors individually, so that adding a general extra-margin for tumor motion (internal margin) is not necessary. Furthermore, we consider a margin for microscopic spread from GTV to the clinical target volume (CTV) in high dose radiotherapy dispensable. Giraud et al report 95% of microscopic tumor spread within a distance of 8 and 6 mm from the gross tumor in adenocarcinomas and squamous cell carcinomas of the lung, respectively [20]. Applied to the presented six patients' gross tumor dose of 81,9 Gy a sufficient dose to the rim of microscopic disease (about 45 Gy in 2,5 weeks) is delivered anyway.

It has been criticized that 4D planning CTs depict more exactly the extreme positions of moving tumors and deliver sharper contours compared to slow CTs. Perhaps this is also a question of institutional practice and habits. Not capturing extreme, short lasting positions of parts of the tumor can be advantageous, when a resulting smaller PTV enables raising the total dose. Also, in handling with somewhat blurred contours drawing the PTV, with some practice we don't see any problem. Summing up, we consider slow planning CTs a simple, effective, non-expensive method, capable to depict the relevant positions of a moving lung tumor.

The issue of setting the junction plane within macroscopic disease has been discussed in our previous report [3]. In the phantom a homogeneously irradiated volume is proven. Actually, with non-splitting techniques there is the same situation: to the patient is offered a homogeneously treated volume. Also, intensity modulated treatments use a multitude of single static and/or dynamic elements resulting in homogeneously treated volumes.

Our planning system facilitates a pencil beam algorithm. More advanced algorithms such as superposition-convolution methods would compute the influence of inhomogeneities on dose distributions more accurately, but this seems to be negligible for the aim of this report.

Target splitting has first enabled the secure application of doses up to 94,5 Gy with conventional fractionation for NSCLC patients [18]. After a phase I/II trial, showing good tolerability of accelerated, twice daily applied high dose radiotherapy in 30 patients, currently a prospective accelerated high dose trial is ongoing, relating the dose to the size of the primary tumors (4 groups: <2,5 cm/73,8 Gy; 2,5–4,5 cm/79,2 Gy; 4,5–6,0 cm/84,6 Gy; >6,0 cm/90,0 Gy; 1,8 Gy bid). The first results in 102 patients show an actuarial local tumor control at 2 years of 82% and an encouraging median overall survival time of 28,0 months [4,19]. Recently, sophisticated forms of intensity modulated techniques such as tomotherapy, intensity modulated arc therapies or volumetric modulated arc therapies have been described [1,2]. As results of treatments of lung cancer patients with these techniques are still missing, a comparison of the efficacy of the different approaches is not yet possible.

Recently, a shift in the incidence from central to peripheral tumors in lung cancer patients has been observed [21]. With its ability to differentiate the beam arrangements, the technique of target splitting seems to be a useful tool especially for peripheral tumors in advanced stages.

With growing incidence we use this technique also for extrathoracic tumor sites, such as thyroid, stomach, pelvic/paraaortic, limbs etc.

Summarizing, the technical developments of target splitting evolved since the first report enable secure dose escalations above 90 Gy for patients with advanced NSCLC, without heavy inroad on resources in term of staff and linac time.

## Competing interests

The authors declare that they have no competing interests.

## Authors' contributions

KW mainly conceived and drafted the manuscript, participated in the conception of target splitting; HD conceived the QA program, drafted its description in the manuscript and gave substantial support in the development of target splitting; PK, FM and HS acquired the data and drafted the figures; FS gave final approval of the version to be published. All authors read and approved the final manuscript.
